# IP3R2-Mediated Astrocytic Ca^2+^ Transients Are Critical to Sustain Modulatory Effects of Locomotion on Neurons in Mouse Somatosensory Cortex

**DOI:** 10.3390/cells14141103

**Published:** 2025-07-18

**Authors:** Mario Fernández de la Puebla, Xiaoyi Zhang, Erlend A. Nagelhus, Magnar Bjørås, Wannan Tang

**Affiliations:** 1 Department of Clinical and Molecular Medicine, Norwegian University of Science and Technology (NTNU), 7491 Trondheim, Norway; mario.f.de.l.puebla@ntnu.no (M.F.d.l.P.); magnar.bjoras@ntnu.no (M.B.); 2 Department of Molecular Medicine, Institute of Basic Medical Sciences, University of Oslo, 0372 Oslo, Norway; caojiezhim@gmail.com; 3 Department of Microbiology, Oslo University Hospital and University of Oslo, 0372 Oslo, Norway

**Keywords:** astrocytes, IP3R2, neuro–glial interaction, Ca^2+^ signaling, two-photon imaging, genetically encoded calcium indicator, locomotion, neuromodulation

## Abstract

Accumulating studies have shown that astrocytes are essential for regulating neurons at both synaptic and circuit levels. The main mechanism of brain astrocytic intracellular Ca^2+^ activity is through the release of Ca^2+^ via the inositol 1,4,5-trisphosphate receptor type 2 (IP3R2) from the endoplasmic reticulum (ER). Studies using IP3R2 knockout mouse models (*Itpr2*^−/−^) have shown that eliminating IP3R2 leads to a significant reduction in astrocytic Ca^2+^ activity However, there is ongoing controversy regarding the effect of this IP3R2-dependent reduction in astrocytic Ca^2+^ transients on neuronal activity. In our study, we employed dual-color two-photon Ca^2+^ imaging to study astrocytes and neurons simultaneously in vibrissa somatosensory cortex (vS1) in awake-behaving wild-type and *Itpr2*^−/−^ mice. We systematically characterized and compared both recorded astrocytic and neuronal Ca^2+^ activities in wild-type and *Itpr2*^−/−^ mice during various animal behaviors, particularly during the transition period from stillness to locomotion. We report that vS1 astrocytic Ca^2+^ elevation in both wild-type and *Itpr2*^−/−^ mice was significantly modulated by free whisking and locomotion. However, vS1 neurons were only significantly modulated by locomotion in wild-type mice, but not in *Itpr2*^−/−^ mice. Our study suggests a non-synaptic modulatory mechanism on functions of astrocytic IP3R2-dependent Ca^2+^ transients to local neurons.

## 1. Introduction

Recent research has reshaped our understanding of astrocytes, revealing them not only as passive support cells but also as active regulators of neural circuits, a role closely tied to their dynamics of intracellular calcium (Ca^2+^) activity [1,2,3,4,5,6,7]. A main mechanism of this astrocytic Ca^2+^ activity is through the release of Ca^2+^ from the endoplasmic reticulum (ER) via the inositol 1,4,5-trisphosphate receptor type 2 (IP3R2), which is highly expressed in astrocytes [8,9]. Studies using IP3R2 knockout mouse models (*Itpr2*^−/−^) have shown that eliminating IP3R2 leads to a significant reduction in astrocytic Ca^2+^ activity [10,11,12,13]. While the loss of IP3R2 markedly diminishes Ca^2+^ transients, some investigations have reported that these signals are not completely abolished, suggesting that additional mechanisms, such as other IP_3_ receptor subtypes or plasma membrane Ca^2+^ entry, contribute to astrocytic Ca^2+^ dynamics [14,15,16,17].

Meanwhile, the impact of the reduced astrocytic Ca^2+^ transients on neuronal function and animal behavior has generated considerable debate. Some reports indicate that diminishing astrocytic Ca^2+^ transients through IP3R2 deletion has little effect on neuronal excitability or behavioral performance [10,11,18,19]. In contrast, other studies have observed alterations in neural circuit dynamics and behavioral deficits in *Itpr2*^−/−^ mice [20,21,22,23,24,25,26]. These contrasting findings raise important questions about how the decrease in astrocytic Ca^2+^ transients might influence the activity of neurons and whether compensatory mechanisms exist to maintain neural circuit stability. However, when studying the reduced astrocytic Ca^2+^ transients in *Itpr2*^−/−^ mice, previous reports have had their conclusion from various ex vivo and in vivo experimental models, and their methodologies and observations differ. It is important to systematically revisit this controversy to review activities from both astrocytes and neurons simultaneously, particularly under awake, freely moving conditions. Furthermore, accumulating evidence reports that astrocytes play a role in behavior or brain state transitions, such as during arousal. These studies indicated the contribution of neuromodulatory systems to astrocytic Ca^2+^ transients [5,6,27]. It is still unclear how IP3R2-induced astrocytic Ca^2+^ may influence the activity of the neurons during these transitions.

In our study, we employed dual-color two-photon Ca^2+^ imaging to study astrocytes and neurons simultaneously in the vibrissa somatosensory cortex (vS1) in head-fixed, awake-behaving wild-type and *Itpr2*^−/−^ mice. Genetically encoded Ca^2+^ indicators, GCaMP6f [28] and jRGECO1a [29], were used to reveal astrocytic and neuronal Ca^2+^ activities, respectively. This allowed us to explore both neurons and astrocytes when IP3R2 is absent. We recorded Ca^2+^ activities in astrocytes and neurons in vS1 under various behavioral states, including stillness (Still), active free whisking (Still-Whisking), and locomotion (Locomotion). We also systematically characterized and compared both recorded astrocytic and neuronal Ca^2+^ activities in wild-type and *Itpr2*^−/−^ mice, particularly during the transition period from stillness to locomotion. To investigate behavior impacts on astrocytic and neuronal Ca^2+^ elevation, we implement a free whisking modulatory index (WhiskMI) and a locomotion modulatory index (LocMI). Our study revealed significant modulatory effect changes of locomotion on neurons in *Itpr2*^−/−^ mice, suggesting a non-synaptic modulatory mechanism on functions of astrocytic IP3R2-dependent Ca^2+^ transients to local neurons.

## 2. Material and Methods

### 2.1. Animals

Male wild-type (C57BL/6J, Janvier Labs) and *Itpr2*^−/−^ (*Itpr2*^tm1.1Chen^; MGI:3640970) mice with the age of 8–12 weeks old were housed with a 12 h light/dark cycle (light on at 8 a.m.) with running wheels and cardboard houses to enrich the housing environment. All animal handling and experimental procedures were performed according to the European Animal Research law (Directive 2010/63/EU) and approved by the Norwegian Food Safety Authority (project number: FOTS 27090).

### 2.2. Plasmid Construction and Virus Production

The DNA sequences for the genetically encoded fluorescent Ca^2+^ indicator GCaMP6f [28] and jRGECO1a [29] were first amplified by PCR from pGP-CMV-GCaMP6f and pGP-CMV-NES-jRGECO1a (Addgene) with 5′ BamHI and 3′ HindIII, and sub-cloned into the recombinant adeno-associated virus (rAAV) vector pAAV-6P-SEWB [30] for generating pAAV-*SYN*-GCaMP6f and pAAV-*SYN*-jRCaMP1a, respectively. The human glial fibrillary acidic protein (*GFAP*) promoter [31] was inserted with MluI and BamHI into the pAAV-*SYN*-GCaMP6f construct for obtaining pAAV-*GFAP*-GCaMP6f. Serotype 2/1 rAAVs from pAAV-*GFAP*-GCaMP6f and pAAV-*SYN*-jRCaMP1a were produced and purified by AVB Sepharose affinity chromatography [32], following titration with real-time PCR (rAAV titer about 1.0–6.0 × 10^12^ viral genomes/mL, TaqMan Assay, Applied Biosystems). For cortical rAAV-transduction of both cell types, the above-produced viruses were mixed 1:1.

### 2.3. Surgical Procedures and Virus Transduction

Mice were briefly anesthetized with 3% isoflurane in an induction chamber and then were quickly fastened on a stereotaxic frame with a nose cone (Model 963, KOPF Instruments, California, CA, USA). Animals were maintained under anesthesia with 1.2–1.5% isoflurane in a mixture of 80% oxygen and 20% room air and buprenorphine 0.1 mg/kg s.c. preemptively for analgesia. Before the surgery, Bupivacaine was administered subcutaneously over the skull and left for 10 min. Skin and connective tissues over the somatosensory cortex were removed. A custom-made titanium head-bar was glued to the mouse skull using cyanoacrylate glue (Loctite super glue) and sealed with dental cement (Meliodent, Kulter). A 2.5-mm-diameter craniotomy was drilled with its center coordinates relative to Bregma of anteroposterior (AP) −1.1 mm and mediolateral (ML) −3.5 mm. A fine dental drill was used to carefully cut the skull with intermittent soaking and removal of debris until only ~0.1 mm of the bone thickness was left. After 10 min of soaking in 0.9% saline, the bone flap was carefully removed. The rAAV mixture was slowly injected (20–30 nL/minute) at three evenly spaced locations (70 nL each at 400 μm depth) positioned to stay clear from large blood vessels. A glass plug consisting of two coverslips with a diameter of 2.5 mm and 3.5 mm glued together with ultraviolet curing glue (Norland Optical Adhesive No. 61) was placed and fixed on the craniotomy using cyanoacrylate-gel glue (Loctite-super glue gel) and reinforced with dental cement (Meliodent, Kulter). The glass window was slightly pressing the dura to prevent dural overgrowth. Mice with implant complications were excluded from further experiments.

### 2.4. Animal Training

Mice were housed in an enriched environment with a freely spinning wheel in their home cages. One week before imaging, mice were habituated to be handled and head-fixed on a freely spinning wheel under the two-photon microscope for increasing durations ranging from 5 min on the first day to 30 min on the last. Mice that showed signs of stress and did not accommodate head restraint were not included in the study.

### 2.5. Two-Photon Imaging

Three to seven weeks after the surgery, mice were imaged under a two-photon microscope (Ultima IV, Bruker/Prairie Technologies) with a 16 ×  0.8 NA water-immersion objective (model CFI75 LWD 16XW, Nikon, Tokyo, Japan), an InSight DeepSee laser (Spectra-Physics) and Peltier-cooled photomultiplier tubes (model 7422PA-40, Hamamatsu Photonics K.K). The excitation wavelength was 990 nm to record both GCaMP6f and jRCaMP1a signals. The optical filters mentioned in the following description of the two-photon microscope are by Chroma Technology Corporation: after the collected light is reflected towards the detection unit by the primary dichroic filter (ZT473-488/594/NIRtpc), the signal light enters the detector house (four channels), passing a ZET473-488/594/NIRm filter, that is shielding the photomultiplier tubes from reflective light. Inside the detector house, the light is split into two fractions separated at a wavelength of 560 nm by a dichroic filter (T560lpxr). The green light (GCaMP6f) is further guided by a secondary dichroic beam splitter at 495 nm (T495lpxr) and filtered by an ET525/50m-2p bandpass filter, whereas a secondary beam splitter similarly directs the red light (jRGECO1a) at 640 nm (T640lpxr) and subsequently filtered by an ET595/50m-2p bandpass filter. Finally, the emitted photons were detected with Peltier-cooled photomultiplier tubes (model 7422PA-40 by Hamamatsu Photonics K.K.). Field of views (FOVs) of ~200 × 200 µm with a resolution of 512 × 512 pixels were acquired at 10 Hz in layer 2/3 of vS1 (focal depth: 175–240 µm), with a recording duration of 5–10 min.

### 2.6. Image Pre-Analysis

Imaging raw data were corrected for motion artifacts using the NoRMCorre movement correction algorithm [33]. To improve the signal-to-noise ratio (SNR) and preserve fine spatiotemporal details in the raw imaging data, we applied the SUPPORT algorithm for denoising. SUPPORT is a self-supervised denoising framework that exploits the inherent spatiotemporal redundancy of two-photon recordings to effectively reduce photon shot noise without requiring ground-truth images for training [34]. The denoised images were subsequently used for region-of-interest (ROI) selection and quantification of fluorescence changes associated with astrocytic and neuronal activity.

### 2.7. Calcium Data Analysis

To analyze calcium changes, ROIs were manually drawn from the mean image of astrocyte and neuron channels. For astrocytes, ROIs were classified by visual morphology identification into four classes: astrocytic somata (AS), astrocytic processes (AP), gliopil (Gp), and astrocytic endfeet (AE). AEs correspond to signals found around the blood vessels, APs were drawn in processes approximately 2–5 µm from the AS, once the main process is subdivided, and Gp was marked in fine processes further away from the AS and AP. Neuron somata (neurons) were selected in the neuron channel by manually drawing ROIs around the doughnut-shaped somas.

Negative drifts (i.e., photobleaching) were corrected by removing the fluorescence trace linear trend (first-degree polynomial) from the raw trace. Relative fluorescence changes were defined with the formula Δ*F*/*F*_0_  =  (*F* – *F*_0_)/*F*_0_, where the baseline (*F*_0_) corresponds to the 10th percentile of the smoothed fluorescence trace (*F*) (5 s window, first-order Savitsky–Golay filter). Neuropil signals around the neuron somas (NS-np) were calculated from 7-μm-wide regions around the neuron soma, avoiding other NSs, and subtracted from neuron somas signals as in *F*_NS_  =  *F*_NS_ − α *F*_NS-np_, where α corresponds to the neuropil contamination ratio, α = 0.7, as previously described [35].

To detect Ca^2+^ events in astrocytes and neurons, baseline noise, *σ*, was defined as the standard deviation of the fluorescence change during the least noisy 5 s period of the fluorescence trace. Ca^2+^ events were detected when the increase of the Δ*F*/*F*_0_ was more prominent than 2- or 2.5-times *σ* for astrocytes and neurons, respectively. The duration of the events was defined as the time points where the Δ*F*/*F*_0_ crossed 0, and the amplitude was the peak of the Δ*F*/*F*_0_ within the duration.

In addition to custom event detection, neuronal spike probabilities were estimated by deconvolving Ca^2+^ traces using CASCADE, a Python toolbox (version 3.7) that employs supervised deep neural networks trained on a large ground-truth dataset [36]. The pretrained model used was “Global_EXC_10Hz_smoothing50ms_causalkernel”.

### 2.8. Behavior Segmentation

Mouse behavior was categorized into three distinct states: ‘Still’, ‘Locomotion’, and ‘Still-Whisking’. Classification was based on movement speed and whisker activity. ‘Locomotion’ periods were defined as time points where forward movement exceeded 1 cm/sec, as measured by a rotary encoder. Brief interruptions in locomotion lasting less than 0.5 s were merged with adjacent ‘Locomotion’ segments. Periods with no movement or with minimal, inconsistent movement below 1 cm/s were labeled as ‘Still’, and short interruptions within these periods (<0.5 s) were also merged.

To calculate the modulatory index, whisking was assessed only during ‘Still’ periods, since mice typically whisk continuously while running. Whisker activity was quantified from infrared video recordings by computing the mean absolute pixel difference between consecutive frames within ROIs drawn over the whisker pad. The resulting trace was smoothed using a moving average filter with a 0.33 s window. To detect whisking, a threshold was applied based on the baseline activity level, defined as the first percentile of the trace (W_0_). Time points were classified as whisking when the smoothed signal exceeded this baseline by more than 1.5. Binarized whisking segments shorter than 0.4 s were excluded, and gaps shorter than 0.3 s between whisking bouts were merged. Periods classified as ‘Still’ that coincided with whisker movement were labeled as ‘Still-Whisking’.

### 2.9. Effect of Free Whisking and Locomotion

Event amplitudes were used to assess the effects of behavior on neurons and astrocytes’ Ca^2+^ signals. As an alternative approach, we implemented a Locomotion Modulatory Index, LocMI, as similarly described previously [37,38]:

(1) $$LocMI=(S_{loc}-S_{s})\;/\;(S_{loc}\;+\;S_{s})$$

*S_loc_* indicates the mean Δ*F*/*F*_0_ value during locomotion periods, and *S_s_* indicates the mean Δ*F*/*F*_0_ value during still segments. Only moving periods > 3 s were included in *S_loc_*. *S_s_* segments were filtered by removing 2 s before and after any locomotion period to avoid including locomotion-related activity. Only 2 s minimum *S_s_* segments after filtering were considered.

Similarly, a Whisking Modulatory Index, WhiskMI, was calculated to account for the effect of free whisking during the Still-Whisking period on Ca^2+^ signals.

(2) $$WhiskMI\;=\;(S_{w}\;-\;S_{s})\;/\;(S_{w}\;+\;S_{s})$$

*S_w_* indicates the mean Δ*F*/*F*_0_ value during whisking periods, and *S_s_* is the same as in LocMI. Only *S_w_* segments of 0.4 s minimum length were considered. Whisking during locomotion could not be assessed as it was impossible to separate the effect of running and free whisking.

### 2.10. Calculation of Ca^2+^ Event Onsets During Still to Locomotion Transition

To quantify the lag of Ca^2+^ event onset relative to locomotion onset, segments spanning from 2 s before to 7 s after the transition were analyzed. Only locomotion periods lasting ≥ 3 s were considered. ROIs with ongoing events before locomotion onset or with Ca^2+^ signals returning to baseline within 1 s before the transition were excluded. Onset lag was estimated via bootstrapping at mouse and ROI levels to account for within mouse and ROI variability.

### 2.11. Calculation of Activation Rate and Response Reliability

Activation rate was defined as the fraction of ROIs activated per transition. Response reliability (RR) to locomotion onset was computed as follows:

(3) $$RR=\frac{RA}{n}$$

where *RA* is the number of transitions during which the ROI exhibited a Ca^2+^ event, and *n* is the total number of transitions in which the ROI was present. Only ROIs with ≥3 transitions were included in the analysis.

### 2.12. Statistical Analysis

To accommodate the hierarchical structure of the data, with multiple observations nested within ROIs and individual mice, we employed linear mixed effects (LME) models. This approach allowed us to simultaneously account for fixed effects (e.g., behavior state, genotype, and their interactions) and random effects (individual mice).

For event amplitude, duration and spike rate LME models were fitted using the nlme package in R [39]. The fixed effects included behavior state (Still, Still-Whisking, or Locomotion), animal group (wild-type or *Itpr2*^−/−^), and their interactions, with each ROI compartment analyzed independently. Individual mice were incorporated as random predictors, influencing the fixed effect of behavior. For the response variables of event amplitude and duration, log-transformed responses were assumed to follow an ordinary linear mixed effects model. Model assumptions were evaluated using residual plots, and in cases where deviations from constant residual variance were observed, the models were extended to allow the residual variance to vary as a function of genotype and state.

ROI event frequency was analyzed using a two-part mixed-effects model fitted with the GLMMadaptive package to address the non-negligible probability of observing zero events [40]. One component of the model estimated the probability of an observation being zero, while the other modeled the non-zero observations using a log-normal distribution. This model utilized the same fixed and random effects structure as described above. Reported *p*-values are based on the t-distribution with degrees of freedom provided by the nlme package. Post hoc pairwise comparisons of the different genotype/behavior combinations were obtained using estimated marginal means (emmeans package).

For the WhiskMI, LocMI, and the onset lag to locomotion, we employed a hierarchical bootstrap to account for the nested structure of our data (mice and ROIs) [41]. Specifically, 10,000 bootstrap samples were generated by resampling with replacement at both levels, providing robust estimates of the modulatory index and its uncertainty. To compare animal groups (wild-type vs. *Itpr2*^−/−^), for each bootstrap iteration, the modulatory index and event onset lag were calculated for both groups and constructed a joint probability distribution of the bootstrap sample means. By calculating the cumulative density on one side of this line, we obtained a direct estimate (*p*_boot_) of the probability that one genotype’s estimate is greater than the other one. Statistical significance was inferred based on whether this probability exceeded the threshold corresponding to our chosen false positive rate (α = 0.05).

All bar plots and boxplots show the mean ± sem unless otherwise stated. The *p*-values (*p*) derived from mixed models and *p*_boot_ derived from hierarchical bootstrapping are indicated as follows: *, *p* < 0.05; **, *p* < 0.01; ***, *p* < 0.001. All comparisons are performed at the animal group level. If there is no level of significance present, it indicates that the difference is not significant (ns, *p* ≥ 0.05).

## 3. Results

### 3.1. Two-Photon Ca^2+^ Imaging Revealed Distinct Astrocytic Activity Patterns in Wild-Type and Itpr2^−/−^ Mice

**Figure 1:**
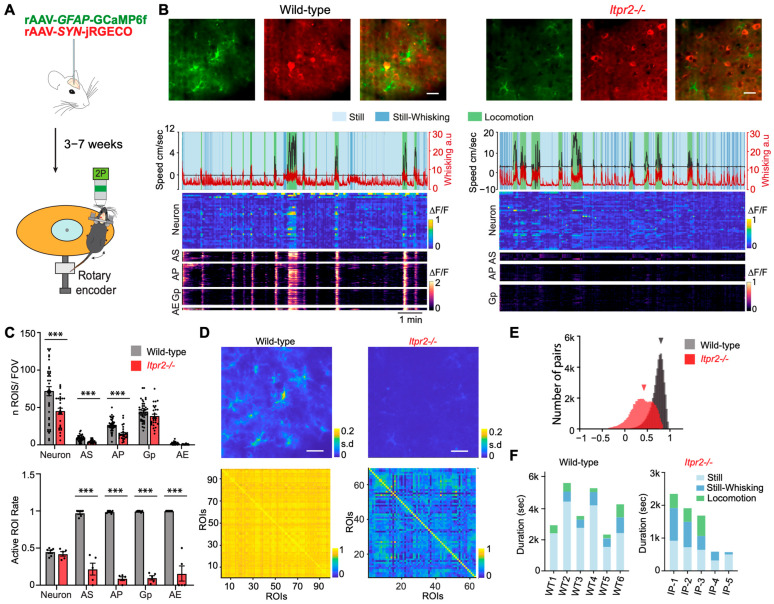
**Experimental design and astrocyte activity differences in wild-type and *Itpr2*^−/−^ mice.** (**A**) Illustration of experimental settings. (**B**) Representative two-photon dual-color imaging and data presentation in wild-type (left) and *Itpr2*^−/−^ mice (right). Top: Average projection images of the field-of-view (FOV) display astrocytes (green), neurons (red), and their merged channels. Below: segmentation of mouse behavioral states (Still, Still-Whisking, and Locomotion) along with corresponding Ca^2+^ signals, which are shown as heatmaps for neurons, and the different astrocytic microcompartments, soma (AS), processes (AP), gliopil (Gp), and endfeet (AE). Scale bar: 20 µm. (**C**) Quantitative comparison of number of ROIs per FOV (top) and active ROI rate with at least one event per recording (bottom). Top: AS: wild-type: 8.66 ± 0.62, *Itpr2*^−/−^: 4.32 ± 0.40, *p* < 0.001; AP: wild-type: 26.66 ± 1.34, *Itpr2*^−/−^: 14.96 ± 1.66, *p* < 0.001; Gp: wild-type: 44.39 ± 2.07, *Itpr2*^−/−^: 38.43 ± 2.68, *p* = 0.0614; AE: wild-type: 1.24 ± 0.32, *Itpr2*^−/−^: 0.57 ± 0.12, *p* = 0.1568. Bottom: AS: wild-type 0.97 ± 0.02, *Itpr2*^−/−^ 0.21 ± 0.08, *p* = 0.0073; AP: wild-type 0.99 ± 0.01, *Itpr2*^−/−^ 0.09 ± 0.025, *p* = 0.008; Gp: wild-type 0.99 ± 0.001, *Itpr2*^−/−^ 0.10 ± 0.029, *p* = 0.0075; AE: wild-type 1.00 ± 0.00, *Itpr2*^−/−^ 0.16 ± 0.10, *p* = 0.0014. Data presented as mean ± sem, *p*. (**D**) Top, standard deviation projection images of the astrocyte channel of wild-type (left) and *Itpr2*^−/−^ mice (right); same FOVs as shown in (**B**). Scale bars: 20 µm. Bottom, activity correlation of astrocytic pairs from ROIs extracted from the FOV shown above. (**E**) Distribution of activity correlations between astrocytic ROI pairs across the entire population of all wild-type and *Itpr2*^−/−^ mice (wild-type r: 0.72 ± 0.14 (mean ± sem) across 139,087 pairs of astrocytes from 41 sessions and six mice; *Itpr2*^−/−^ r: 0.43 ± 0.25 (mean ± sem) across 52,540 pairs of astrocytes from 28 sessions and five mice; *p*_boot_ < 0.001). (**F**) Total duration of each behavioral state recorded from all mice: 48 recordings (10 min each) from six wild-type mice and 21 recordings (5–10 min each) from five *Itpr2*^−/−^ mice.; ***, *p* < 0.001.

To simultaneously study both astrocytic and neuronal activities, we performed the dual-color two-photon imaging in both astrocytes and neurons using GCaMP6f and jRGECO1a, respectively in head-fixed freely moving mice. To access the natural whisking and movement behaviors in these animals, we choose vS1 as our regions of interest for recording (Figure 1A). Both wild-type and *Itpr2*^−/−^ mice were imaged, and Ca^2+^ dynamics during different behavioral states were recorded. According to animal behaviors, all recordings were segmented into three behavior categories: Locomotion (speed > 1 cm/s and duration > 0.5 s), Still (speed < 1 cm/s and duration > 0.5 s, details in Section 2), and Still-Whisking (Still periods with concomitant free whisking duration > 0.4 s). Figure 1B shows example traces of the behavioral monitoring and segmentation, as well as calcium imaging in astrocytes and neurons from recorded wild-type and *Itpr2*^−/−^ mice.

It has been reported that different astrocytic subcellular microcompartments present distinct Ca^2+^ signal properties [14,42,43], namely the cell body/soma (AS), processes (AP), gliopil (Gp, fine processes further away from the AS and AP), and endfeet (AE) [44,45]. Next, across 41 field of views (FOVs) from six wild-type mice and 28 FOVs from five *Itpr2*^−/−^ mice, we manually marked all astrocytic microcompartments and neurons (cell body) to analyze their Ca^2+^ transients within these regions of interest (ROIs). In all types of astrocytic ROIs, *Itpr2*^−/−^ mice exhibited a significant reduction in the number of astrocytic ROIs in AS and AP when compared to wild-type mice (Figure 1C, upper panel, AS, *p* < 0.001; AP, *p* < 0.001; Gp, *p* = 0.0614; AE, *p* = 0.1568). This confirms that the deletion of IP3R2 alters the detectable Ca^2+^ signals in astrocytes. Interestingly, we also observed a reduction in neuronal ROIs (*p* = 0.0024).

We further quantified the proportion of active ROIs (exhibiting at least one Ca^2+^ event during the recording session) among all marked ROIs. In wild-type mice, a substantial fraction of astrocytic ROIs was active across all conditions, whereas in *Itpr2*^−/−^ mice, the proportions of all astrocytic active ROIs were significantly lower (Figure 1C, lower panel, AS, *p* = 0.0073; AP, *p* = 0.008; Gp, *p* = 0.0075; AE, *p* = 0.0014). Regardless of the above-observed lower number of marked neurons in *Itpr2*^−/−^ mice when compared to the wild-type mice, the proportion of active neuronal ROIs did not show any difference (*p* = 0.99). This finding aligns with the previously reported data that the absence of IP3R2 in astrocytes decreases the likelihood of Ca^2+^ transients in all astrocytic microcompartments, but not in neurons [19]. The above data can be also visualized in the representative standard deviation projection images shown in Figure 1D. The wild-type FOV displays prominent Ca^2+^ activity, while in the *Itpr2*^−/−^ FOV, astrocytic Ca^2+^ transients appear dimmer, indicating reduced signal transients. In addition, pairwise correlation of Ca^2+^ ROIs in wild-type and *Itpr2*^−/−^ mice further illustrates these differences (Figure 1E). Astrocytic ROIs show strong, widespread correlations in wild-type mice, whereas in *Itpr2*^−/−^ mice, correlation values are lower and more variable (Figure 1F, *p*_boot_ < 0.001). These results suggest that IP3R2 deletion severely weakens coordinated astrocytic activity.

To compare the differences of Ca^2+^ transients in wild-type and *Itpr2*^−/−^ mice during various behavioral states, we quantified the time spent in Still, Still-Whisking, and Locomotion (Figure 1F, six wild-type and five *Itpr2*^−/−^ mice). Both wild-type and *Itpr2*^−/−^ mice exhibited all behavioral states, with only two *Itpr2*^−/−^ mice failing to engage in Locomotion. While there were variations in the relative time spent in each state, no clear genotype-dependent difference in behavioral distribution was observed. This suggests that, despite the loss of IP3R2, mice were capable of engaging in all behaviors, though potential subtle differences in state transitions or movement dynamics were observed.

**Figure 2:**
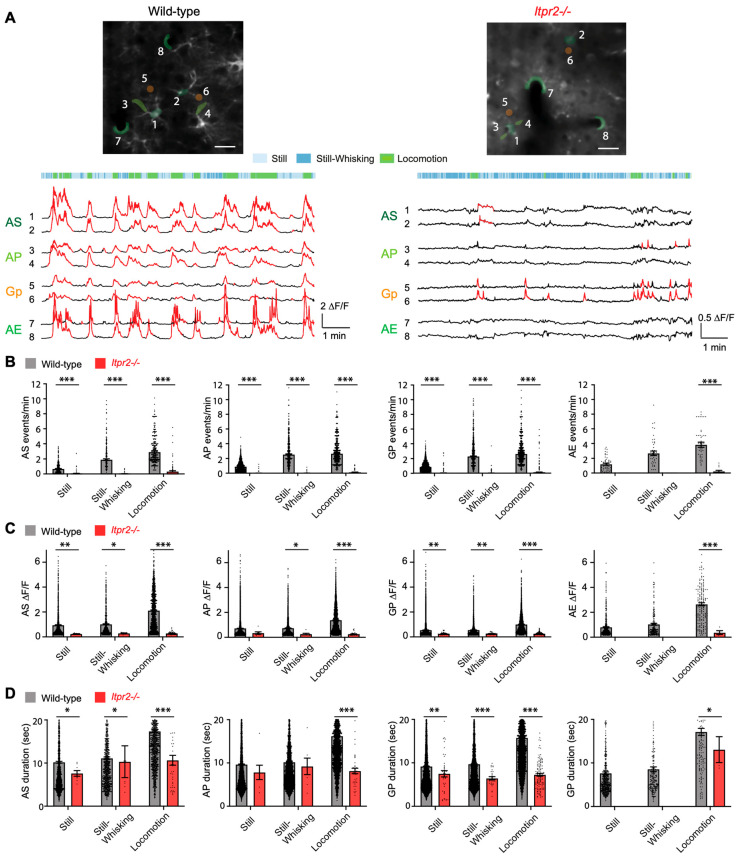
**Layer 2/3 vS1 astrocytes showed reduced Ca^2^^+^ activity in *Itpr2*^−/−^ mice.** (**A**) Top, representative images of astrocytes with ROIs marked with 1–8 from all four astrocytic microcompartments indicated in various colors, in wild-type (left) and *Itpr2*^−/−^ mice (right). Bottom, representative ROI Δ*F*/*F* traces from ROI 1-8 in the above images together with behavioral segmentation in recording timeline. Red traces highlight detected Ca^2+^ events. Soma (AS), processes (AP), gliopil (Gp), and endfeet (AE). Scale bar: 20 µm. (**B**–**D**) Measurement of Ca^2+^ event parameters across different behavioral states (Still, Still-Whisking, and Locomotion). (**B**) Comparison of astrocytic event frequency. Still: AS, wild-type: 0.69 ± 0.03 (355), *Itpr2*^−/−^: 0.06 ± 0.03 (110), *p* < 0.0001; AP, wild-type: 0.87 ± 0.02 (1093), *Itpr2*^−/−^: 0.01 ± 0.004 (381), *p* < 0.0001; Gp, wild-type: 0.86 ± 0.01 (1820), *Itpr2*^−/−^: 0.03 ± 0.00 (1026), *p* < 0.0001; AE, wild-type: 1.20 ± 0.14 (51), *Itpr2*^−/−^: 0 (16), *p* = —. Still-Whisking AS, wild-type: 1.92 ± 0.11 (308), *Itpr2*^−/−^: 0.03 ± 0.013 (90), *p* < 0.0001; AP, wild-type: 2.56 ± 0.07 (967), *Itpr2*^−/−^: 0.001 ± 0.003 (309), *p* < 0.0001; Gp, wild-type: 2.28 ± 0.05 (1632), *Itpr2*^−/−^: 0.014 ± 0.00 (852), *p* < 0.0001; AE, wild-type: 2.68 ± 0.29 (48), *Itpr2*^−/−^: 0 (13), *p* = —. Locomotion: AS, wild-type: 2.91 ± 0.10 (333), *Itpr2*^−/−^: 0.34 ± 0.09 (113), *p* < 0.0001; AP, wild-type: 2.66 ± 0.06 (1020), *Itpr2*^−/−^: 0.13 ± 0.032 (390), *p* < 0.0001; Gp, wild-type: 2.62 ± 0.04 (1708), *Itpr2*^−/−^: 0.14 ± 0.02 (970), *p* < 0.0001; AE, wild-type: 3.83 ± 0.35 (46), *Itpr2*^−/−^: 0.23 ± 0.13 (14), *p* < 0.0001. (**C**) Comparison of astrocytic amplitude. Still: AS, wild-type: 0.95 ± 0.02 (1864), *Itpr2*^−/−^: 0.24 ± 0.016 (8), *p* = 0.0024; AP, wild-type: 0.72 ± 0.00 (7134), *Itpr2*^−/−^: 0.34 ± 0.1 (7), *p* = 0.188; Gp, wild-type: 0.55 ± 0.00 (11,642), *Itpr2*^−/−^: 0.25 ± 0.01 (41), *p* = 0.0016; AE, wild-type: 0.80 ± 0.04 (478), *Itpr2*^−/−^: 0 (0), *p* = —. Still-Whisking: AS, wild-type: 1.02 ± 0.04 (778), *Itpr2*^−/−^: 0.29 ± 0.027 (8), *p* = 0.0196; AP, wild-type: 0.74 ± 0.01 (3253), *Itpr2*^−/−^: 0.26 ± 0.06 (7), *p* = 0.032; Gp, wild-type: 0.56 ± 0.01 (4954), *Itpr2*^−/−^: 0.26 ± 0.015 (27), *p* = 0.0013; AE, wild-type: 1.04 ± 0.08 (195), *Itpr2*^−/−^: 0 (0), *p* = —. Locomotion: AS, wild-type: 2.12 ± 0.046 (1089), *Itpr2*^−/−^: 0.29 ± 0.022 (38), *p* < 0.0001; AP, wild-type: 1.39 ± 0.02 (3135), *Itpr2*^−/−^: 0.26 ± 0.02 (38), *p* < 0.0001; Gp, wild-type: 1.00 ± 0.01 (4987), *Itpr2*^−/−^: 0.27 ± 0.01 (119), *p* < 0.0001; AE, wild-type: 2.65 ± 0.12 (163), *Itpr2*^−/−^: 0.36 ± 0.15 (4), *p* < 0.0001. (**D**) Comparison of astrocytic duration. Still: AS, wild-type: 10.14 ± 0.21(1864), *Itpr2*^−/−^: 7.56 ± 0.65 (8), *p* = 0.024; AP, wild-type: 9.63 ± 0.09 (7134), *Itpr2*^−/−^: 7.8 ± 1.66 (7), *p* = 0.186; Gp, wild-type: 9.19 ± 0.07 (11,642), *Itpr2*^−/−^: 7.50 ± 0.72 (41), *p* = 0.002; AE, wild-type: 7.51 ± 0.29 (478), *Itpr2*^−/−^: 0 (0), *p* = —. Still-Whisking: AS, wild-type: 11.05 ± 0.30 (778), *Itpr2*^−/−^: 10.26 ± 3.67 (8), *p* = 0.0196; AP, wild-type: 10.16 ± 0.13 (3253), *Itpr2*^−/−^: 9.19 ± 1.90 (7), *p* = 0.0545; Gp, wild-type: 9.72 ± 0.10 (4954), *Itpr2*^−/−^: 6.44 ± 0.38, *p* = 0.0009 (27); AE, wild-type: 8.56 ± 0.54 (195), *Itpr2*^−/−^: 0 (0), *p* = —. Locomotion: AS, wild-type: 17.28 ± 0.29 (1089), *Itpr2*^−/−^: 10.59 ± 1.13 (38), *p* < 0.0001; AP, wild-type: 16.18 ± 0.16 (3135), *Itpr2*^−/−^: 8.12 ± 0.63 (38), *p* < 0.0001; Gp, wild-type: 15.80 ± 0.12 (4987), *Itpr2*^−/−^: 7.27 ± 0.33 (119), *p* < 0.0001; AE, wild-type: 17.12 ± 0.80 (163), *Itpr2*^−/−^: 13.05 ± 2.98 (4), *p* = 0.0125. Data presented as mean ± sem (number of events), *p*. The *p*-values are derived from post hoc two-sided tests with Tukey’s adjustment for multiple comparisons, following a linear mixed-effects model analysis. *, *p* < 0.05; **, *p* < 0.01; ***, *p* < 0.001.

To assess the impact of IP3R2-dependent signaling on astrocytic Ca^2+^ dynamics, we analyzed the astrocytic Ca^2+^ event frequency, the amplitude (ΔF/F), and the duration in wild-type and *Itpr2*^−/−^ mice across different behavioral states. Example Ca^2+^ traces from all astrocytic compartments together with the animal behavior segmentation in both wild-type and *Itpr2*^−/−^ mice are illustrated in Figure 2A. Across all behavior conditions, the event frequency was significantly lower in *Itpr2*^−/−^ compared to wild-type mice, with markedly fewer ROIs and Ca^2+^ events detected (Figure 2B, Still: AS, *p* < 0.0001; AP, *p* < 0.0001; Gp, *p* < 0.0001; Still-Whisking AS, *p* < 0.0001; AP, *p* < 0.0001; Gp, *p* < 0.0001; Locomotion: AS, *p* < 0.0001; AP, *p* < 0.0001; Gp, *p* < 0.0001; AE, *p* < 0.0001). In AE, no ROIs or events were identified in the Still and Still-Whisking conditions, resulting in no statistical comparisons. The Ca^2+^ event amplitude was also significantly lower in *Itpr2*^−/−^ mice across all behavioral states (Figure 2C, Still: AS, *p* = 0.0024; AP, *p* = 0.188; Gp, *p* = 0.0016; Still-Whisking: AS, *p* = 0.0196; AP, *p* = 0.032; Gp, *p* = 0.0013; Locomotion: AS, *p* < 0.0001; AP, *p* < 0.0001; Gp, *p* < 0.0001; AE, *p* < 0.0001). Event duration was consistently reduced in *Itpr2*^−/−^ mice compared to the ones in wild-type during Locomotion, indicating that in addition to the lower event frequency and amplitude, astrocytic Ca^2+^ transients in *Itpr2*^−/−^ mice were also shorter in duration (Figure 2D, Still: AS, *p* = 0.024; AP, *p* = 0.186; Gp, *p* = 0.002; Still-Whisking: AS, *p* = 0.0196; AP, *p* = 0.0545; Gp, *p* = 0.0009; Locomotion: AS, *p* < 0.0001; AP, *p* < 0.0001; Gp, *p* < 0.0001).

### 3.2. The Absence of IP3R2 Reduced Astrocytic Ca^2+^ Transients During the Transition Period from Still to Locomotion

**Figure 3:**
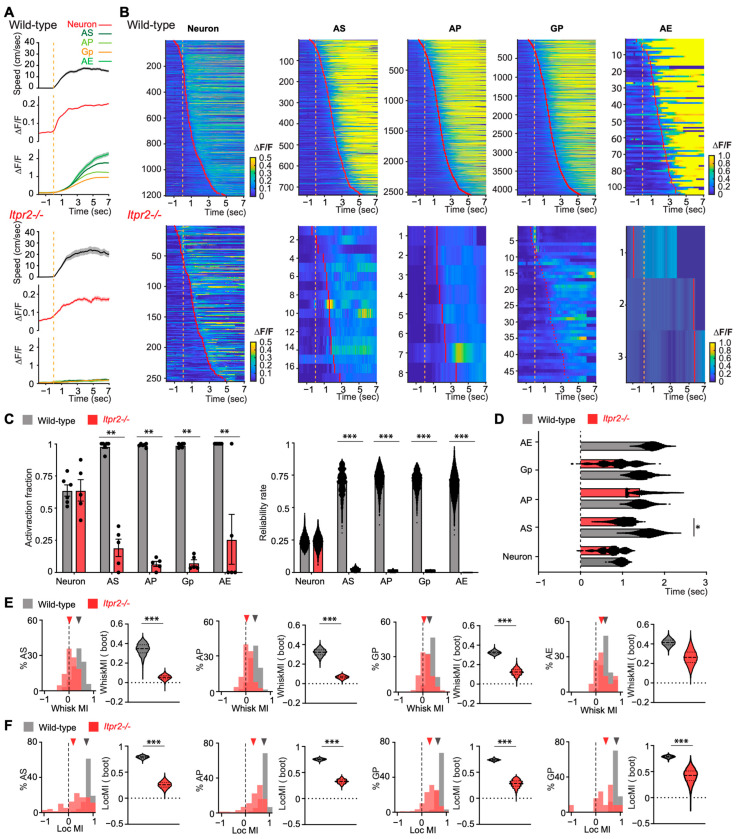
**Ca^2+^ transients in neurons and astrocytes during the transition period from Still to Locomotion** (**A**–**C**)**, and behavioral modulatory effects on astrocytic Ca^2+^ transients** (**E**,**F**). (**A**) Astrocytic microcompartments (Soma (AS), processes (AP), gliopil (Gp), and endfeet (AE)) and neuronal activity are shown during the transition from Still to Locomotion in wild-type and *Itpr2*^−/−^ mice. Average ΔF/F traces (± sem) are color-coded by ROI type and accompanied by the average speed profile on the top (black trances, wild-type: 187 transitions from 40 recordings, six mice; *Itpr2*^−/−^: 128 transitions from 25 recordings, five mice). Vertical dashed line in orange indicates the Locomotion onset. (**B**) Heatmaps of ΔF/F signals for individual events over the transition period. Vertical dashed line in orange indicates the Locomotion onset. Red vertical lines within the heatmap represents event onsets. (**C**) Quantification of active ROI fraction (left, at least one event) and response reliability rate (right, least three instances of the transition) in two animal groups. Left: Neurons, wild-type: 0.64 ± 0.04; *Itpr2*^−/−^: 0.64 ± 0.083; *p* = 0.99; AS, wild-type: 0.98 ± 0.016; *Itpr2*^−/−^: 0.19 ± 0.07; *p* = 0.0073; AP, wild-type: 0.99 ± 0.0054; *Itpr2*^−/−^: 0.07 ± 0.02; *p* = 0.008; Gp, wild-type: 0.99 ± 0.006; *Itpr2*^−/−^: 0.07 ± 0.023; *p* = 0.0075; AE, wild-type: 1.00 ± 0.00; *Itpr2*^−/−^: 0.25 ± 0.19; *p* = 0.0014. Right: Neurons, wild-type: 0.243 ± 0.001; *Itpr2*^−/−^: 0.238 ± 0.0001; *p*_boot_ = 0.548; AS, wild-type: 0.70 ± 0.0001; *Itpr2*^−/−^: 0.02 ± 0.0001; *p*_boot_ < 0.001; AP, wild-type: 0.72 ± 0.0007; *Itpr2*^−/−^: 0.01 ± 0.0001; *p*_boot_ < 0.001; Gp, wild-type: 0.70 ± 0.0006; *Itpr2*^−/−^: 0.01 ± 0.0003; *p*_boot_ < 0.001; AE, wild-type: 0.67 ± 0.001; *Itpr2*^−/−^: –. (**D**) Quantification of event onsets from Still to Locomotion in two animal groups. Plot with bootstrap median ± sem (based on 10,000 samples) for each ROI type. The *p*_boot_ was determined via a bootstrap comparison between two groups (Neuron, wild-type: 1.03 ± 0.01 s; *Itpr2*^−/−^: 0.93 ± 0.002 s; *p*_boot_ = 0.5094; AS, wild-type: 1.63 ± 0.002 s; *Itpr2*^−/−^: 1.043 ± 0.002 s; *p*_boot_ = 0.020; AP, wild-type: 1.44 ± 0.01 s; *Itpr2*^−/−^: 1.45 ± 0.03 s; *p*_boot_ = 0.976; Gp, wild-type: 1.44 ± 0.001 s; *Itpr2*^−/−^: 0.95 ± 0.003 s; *p*_boot_ = 0.195). (**E**) Whisker modulatory index (WhiskMI) for different astrocytic microcompartments in wild-type and *Itpr2*^−/−^ mice. For each microcompartment, left, distribution of WhiskMI; right, violin plots of bootstrap mean WhiskMI derived from 10,000 population samples. The *p*_boot_ value indicates the significance of the two-sided test comparing the two bootstrapped populations. (AS, wild-type: 0.343 ± 0.114 (308), *Itpr2*^−/−^: 0.055 ± 0.050 (93), *p*_boot_ < 0.001; AP, wild-type: 0.320 ± 0.072 (962), *Itpr2*^−/−^: 0.067 ± 0.034 (312), *p*_boot_ < 0.001; Gp, wild-type: 0.322 ± 0.036 (1632), *Itpr2*^−/−^: 0.122 ± 0.072 (831), *p*_boot_ < 0.001; AE, wild-type: 0.414 ± 0.068 (48), *Itpr2*^−/−^: 0.263 ± 0.152 (15), *p*_boot_ = 0.084. (**F**) Locomotion modulatory index (LocMI) for different astrocytic microcompartments in wild-type and *Itpr2*^−/−^ mice. For each microcompartment, left, distribution of LocMI; right, violin plots of bootstrap mean LocMI derived from 10,000 population samples (AS, wild-type: 0.787 ± 0.053 (349), *Itpr2*^−/−^: 0.257 ± 0.126 (83), *p*_boot_ < 0.001; AP, wild-type: 0.752 ± 0.041 (1069), *Itpr2*^−/−^: 0.328 ± 0.084 (292), *p*_boot_ < 0.001; Gp, wild-type: 0.737 ± 0.036 (1780), *Itpr2*^−/−^: 0.281 ± 0.142 (765), *p*_boot_ < 0.001; AE, wild-type: 0.785 ± 0.044 (50), *Itpr2*^−/−^: 0.415 ± 0.266(13), *p*_boot_ < 0.001). Data presented as mean ± sem (number of events), *p*. Colored triangles indicate the median of the observed MI. The *p*_boot_ value indicates the significance of the two-sided test comparing the two bootstrapped populations. *, *p* < 0.05; **, *p* < 0.01; ***, *p* < 0.001.

Examining the temporal dynamics of Ca^2+^ signaling during behavioral transitions can help disentangle the respective contributions of astrocytes and neurons. The transition to locomotion marks a period of heightened arousal and rapidly shifting activity patterns [46,47]. In layer 2/3 of the vS1, which integrates sensory and motor signals [48,49], this state change provides a valuable window to assess whether IP3R2 deletion selectively affects astrocytic Ca^2+^ transients and associated neuronal activity. To investigate this, we analyzed Ca^2+^ transients in both neurons and astrocytes during the transition from Still to Locomotion. We aligned all Ca^2+^ traces to the start of the Locomotion and visualized both the average ΔF/F signals (Figure 3A) and the corresponding Ca^2+^ events as heatmaps (Figure 3B). In wild-type mice, neurons showed rapid activation at Locomotion onset, while astrocytic signals followed (Figure 3A,B, upper panels). In *Itpr2*^−/−^ mice, neuronal responses remained similar; however, the astrocytic activity was strongly reduced, as shown in the near-flat average trace (Figure 3A, lower panel) and the lower number of detected events in the heatmap (Figure 3B, lower panel), indicating a substantial loss of astrocytic responsiveness during locomotion.

Before quantifying Ca^2+^ activity onsets during the transition from Still to Locomotion, we first measured the active fraction of ROIs in both neurons and astrocytes (Figure 3C, left). Neuronal activation did not differ between animal groups (*p* = 0.99). In contrast, the numbers of active astrocytic ROIs were significantly reduced in *Itpr2*^−/−^ mice across all microcompartments, indicating a broad impairment in astrocytic responsiveness during the transition period to Locomotion (AS, *p* = 0.0073; AP, *p* = 0.008; *p* = 0.0075; AE, *p* = 0.0014). We next evaluated the reliability of Ca^2+^ event recurrence across Locomotion transitions (Figure 3C, middle, see Section 2). Neuronal response reliability remained similar in both wild-type and *Itpr2*^−/−^ mice (*p*_boot_ = 0.548), whereas astrocytic reliability was dramatically reduced in all compartments in *Itpr2*^−/−^ mice, suggesting a loss of consistency in astrocytic responses during the transition period to Locomotion (AS, *p*_boot_ < 0.001; AP, *p*_boot_ < 0.001; Gp, *p*_boot_ < 0.001). Due to the lack of active ROIs in AE, the statistical comparison was not performed in AE. Finally, we quantified and compared the Ca^2+^ event onsets relative to Locomotion onsets in both neurons and astrocytes (Figure 3C, right). In neurons, the Ca^2+^ event onsets were similar in both wild-type and *Itpr2*^−/−^ mice (*p*_boot_ = 0.5094). In astrocytes, event onsets in AP were similar in both groups (*p*_boot_ = 0.976); however, the onsets from AS and Gp were faster in *Itpr2*^−/−^ mice than in wild-type mice with a statistical significance in AS (AS, *p*_boot_ = 0.020; Gp, *p*_boot_ = 0.195). Due to the lack of active ROIs in AE, the statistical comparison was not performed in AE. Taken together, these results show that the number of responsive astrocytes and the reliability of their responses in *Itpr2*^−/−^ mice were severely reduced during the transition period to Locomotion. In addition, in *Itpr2*^−/−^ mice, the Ca^2+^ event onsets during Locomotion transition in neurons were not altered; however, they were faster in AS (with significance) and Gp when compared to the ones in wild-type mice (Figure 3D).

These findings suggest that IP3R2-mediated Ca^2+^ signaling in astrocytes may not be required to initiate astrocytic Ca^2+^ transients during the transition to locomotion, as some events with similar onset timing are still observed in the absence of IP3R2. However, IP3R2 may be important for amplifying or sustaining Ca^2+^ transients in astrocytes, which could explain the reduced number of detected events and the overall loss of consistency across transitions. In contrast, neuronal responses appeared overall unaffected, indicating that only astrocytic Ca^2+^ transients are dependent on IP3R2 during the transition to Locomotion.

### 3.3. Itpr2^−/−^ Mice Exhibit Reduced but Not Totally Abolished Astrocytic Ca^2+^ Transients Modulated by Whisking and Locomotion

To further quantify the influence of behavioral states on astrocytic Ca^2+^ transients, we implemented a modulatory index that captures both whisking (WhiskMI, Figure 3E) and locomotion (LocMI, Figure 3F) effects (see Section 2 for details). To calculate the WhiskMI, whisking only referred to the whisker movement during ‘Still-Whisking’ periods. However, during Locomotion, mice typically whisk continuously while moving. Since we did not include any sensory task during the recording, the whisking accessed here is the animal free whisking.

While events were detected using a custom algorithm, the modulatory index was computed from the ΔF/F values, providing a continuous measure of behavior-dependent changes in astrocytic activity. In wild-type mice, both free whisking and locomotion significantly positively modulated astrocytic Ca^2+^ transients in all microcompartments, whereas in *Itpr2*^−/−^ mice, the modulatory effects of whisking and locomotion were reduced (Figure 3E,F, WhiskMI: AS, *p*_boot_ < 0.001; AP, *p*_boot_ < 0.001; Gp, *p*_boot_ < 0.001; AE, *p*_boot_ = 0.084; LocMI: AS, *p*_boot_ < 0.001; AP, *p*_boot_ < 0.001; Gp, *p*_boot_ < 0.001; AE, *p*_boot_ < 0.001). Despite the significantly reduced modulatory effects, after bootstrapping, the WhiskMI and LocMI in *Itpr2*^−/−^ mice remained above zero, indicating that locomotion and whisking behaviors still exerts positive modulatory influence on astrocytic Ca^2+^ transients even when IP3R2 was absent. These results show that although *Itpr2*^−/−^ mice exhibited a profound reduction in their intracellular Ca^2+^ transients, whisking and locomotion still exerted detectable modulatory effects on these reduced Ca^2+^ transients.

### 3.4. Locomotion Failed to Modulate Layer 2/3 vS1 Neurons in Itpr2^−/−^ Mice

**Figure 4:**
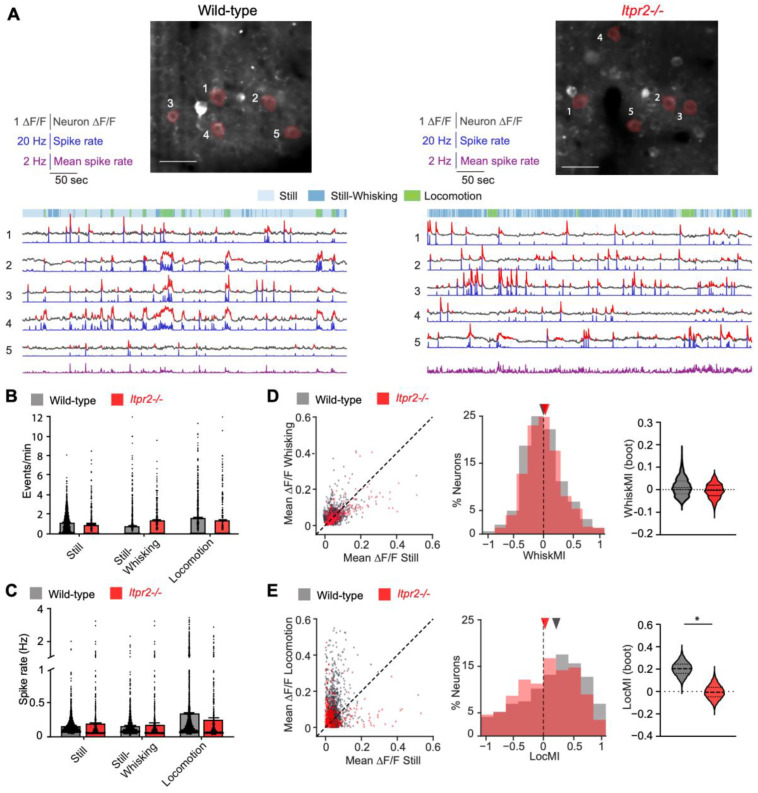
**Behavioral modulatory effects on neuronal Ca^2+^ activity in wild-type and *Itpr2*^−/−^ mice.** (**A**) Top: representative images of neurons with ROIs marked with 1-5 indicated in red in wild-type (left) and *Itpr2*^−/−^ (right) mice. Bottom, representative neuronal ROI 1-5 Δ*F*/*F* traces (black) with detected events (red), deconvolved spike rates (blue), and the mean spike rate across all neurons in the FOV (purple) from the above images together with behavioral segmentation in recording timeline. Scale bars: 40 µm. (**B**) Quantification of neuronal Ca^2+^ event frequency across behavioral states in two mouse groups (Still, wild-type: 1.19 ± 0.03 (1473), *Itpr2*^−/−^: 0.95 ± 0.06 (590), *p* = 0.457; Still-Whisking, wild-type: 0.80 ± 0.04 (1238), *Itpr2*^−/−^: 1.31 ± 0.07 (505), *p* = 0.12; Locomotion, wild-type: 1.62 ± 0.05 (1332), *Itpr2*^−/−^: 1.35 ± 0.09 (561), *p* = 0.096). (**C**) Quantification of neuronal spike rate across behavioral states in two mouse groups (Still, wild-type: 0.168 ± 0.004 Hz(1473), *Itpr2*^−/−^: 0.167 ± 0.01 Hz (596), *p* = 0.724; Still-Whisking, wild-type: 0.145 ± 0.006 Hz (1473), *Itpr2*^−/−^: 0.187 ± 0.01 Hz (630), *p* = 0.279; Locomotion, wild-type: 0.336 ± 0.013 Hz, *Itpr2*^−/−^: 0.24 ± 0.016 Hz (566), *p* = 0.0707). The *p*-values are derived from post hoc two-sided tests with Tukey’s adjustment for multiple comparisons, following a linear mixed-effects model analysis. (**D**) Left: Scatterplots comparing mean ΔF/F during whisking against still periods for individual neurons in wild-type and *Itpr2*^−/−^ mice. Middle: histograms display the distribution of the WhiskMI. Right: violin plots of bootstrap mean WhiskMI derived from 10,000 population samples (wild-type: 0.01 ± 0.08, (1226); *Itpr2*^−/−^: –0.04 ± 0.082, (465); *p*_boot_ = 0.521). (**E**) Left: Scatterplots comparing mean ΔF/F during Locomotion against Still periods for individual neurons in wild-type and *Itpr2*^−/−^ mice. Middle: histograms display the distribution of the LocMI. Right: violin plots of bootstrap mean LocMI derived from 10,000 population samples (wild-type: 0.203 ± 0.115, (1233); *Itpr2*^−/−^: −0.02 ± 0.19, (288); *p*_boot_ = 0.0482). Data presented as mean ± sem (number of events), *p*). Colored triangles indicate the median of the observed MI. The *p*_boot_ represents the significance of the two-sided test comparing the two bootstrapped populations. *, *p* < 0.05.

We have observed a strong reduction in astrocytic Ca^2+^ transients in *Itpr2*^−/−^ mice across behavioral conditions, including weaker modulatory effects from whisking and locomotion. Next, we examined how this reduced astrocytic Ca^2+^ activity would affect local vS1 neurons. To address this, we analyzed Ca^2+^ changes in neurons (ΔF/F) and inferred spike rates by deconvolving Ca^2+^ traces using CASCADE toolbox [36] across different behavioral states. Example traces of Ca^2+^ activity and spike rate from recordings in a wild-type mouse and an *Itpr2*^−/−^ mouse, along with behavioral state segmentation, are shown in Figure 4A.

We first characterized neuronal responses across all behavioral states. Neuronal Ca^2+^ event frequency did not show significant differences between wild-type and *Itpr2*^−/−^ mice, however, with a slight reduction in *Itpr2*^−/−^ mice during Locomotion (Figure 4B, Locomotion, wild-type: 1.62 ± 0.05 (1332), *Itpr2*^−/−^: 1.35 ± 0.09 (561), *p* = 0.096). Similar to event frequency, no significant differences were detected with the inferred spike rates between the two animal groups, however, with a reduction in *Itpr2*^−/−^ mice during Locomotion (Figure 4C, Locomotion, wild-type: 0.336 ± 0.013 Hz, *Itpr2*^−/−^: 0.24 ± 0.016 Hz (566), *p* = 0.0707). In addition, during Locomotion, both event frequency and spike rates were significantly increased when compared to those during Still and Still-Whisking in both animal groups (event frequency, wild-type: Still vs. Locomotion, *p* < 0.001; *Itpr2*^−/−^, Still vs. Locomotion, *p* = 0.044; Spike rate, wild-type, Still vs. Locomotion, *p* < 0.001. *Itpr2*^−/−^, Still vs. Locomotion, *p* = 0.014), indicating in *Itpr2*^−/−^ mice, although slightly reduced during Locomotion, a comparable neuronal activity was observed in vS1.

To assess how behavior modulates vS1 neuronal activity, we first compared the amplitudes of calcium signals during stillness versus free whisking or locomotion in neurons (Figure 4D,E, left). To quantify behavioral modulation on neurons, we calculated WhiskMI, which was centered around zero in both animal groups (Figure 4D, middle); however, the LocMI was shifted positively in wild-type mice, but not in *Itpr2*^−/−^ mice (Figure 4D, middle). Violin plots of bootstrapped WhiskMI values confirmed that whisking had no significant modulatory effect on neurons (Figure 4D, right, *p*_boot_ = 0.521), whereas locomotion (LocMI) significantly enhanced neuronal activity in wild-type but not in *Itpr2*^−/−^mice (Figure 4E, right, *p*_boot_ = 0.0482). Taken together, the above results show that vS1 neurons in wild-type and *Itpr2*^−/−^ mice show similar activity frequency during free whisking and locomotion with a slight reduction in *Itpr2*^−/−^ mice (Figure 4B,C), suggesting a subtle attenuation of neuronal activation in the absence of astrocytic IP3R2 signaling. Furthermore, free whisking did not cause any modulation on vS1 neurons in both animal groups, whereas the modulatory effect of locomotion was significantly reduced in *Itpr2*^−/−^ mice, indicating that the reduced astrocytic Ca^2+^ transients observed in *Itpr2*^−/−^ mice may be critical in sustaining the modulation of locomotion on vS1 neurons.

## 4. Discussion

In this study, we systematically characterized and compared Ca^2+^ activities in both astrocytes and neurons in vS1 in wild-type and *Itpr2*^−/−^ mice during various animal behavior states. Our findings aligned with previous reports on the profound attenuation of astrocytic intracellular Ca^2+^ mediated by IP3R2, however, not completely abolished [14,16,17,50]. Meanwhile, on the neuronal side, we found that the neuronal activity, analyzed by both Ca^2+^ event detection and inferred spike rates, showed no significant differences in both genotypes, although with a slight reduction in *Itpr2*^−/−^ mice. Interestingly, locomotion showed no positive modulation on neurons in *Itpr2*^−/−^ mice, which significantly differed from the modulatory effect in wild-type mice.

We observed robust Ca^2+^ transients in astrocytes across all compartments: soma, processes, and endfeet in wild-type mice, whereas *Itpr2*^−/−^ mice showed a drastic reduction in astrocytic Ca^2+^ events. Notably, as reported before, astrocyte activity was not completely abolished in *Itpr2*^−/−^ mice with persistent small, sporadic Ca^2+^ transients [14,50]. These residual events indicate that astrocytes retain some Ca^2+^ signaling capacity through IP3R2-independent pathways. Indeed, astrocytes express a variety of alternative Ca^2+^ influx and release mechanisms that likely underlie this remaining activity. These include plasmalemmal Ca^2+^ influx through different ionotropic receptors [51,52,53], the sodium/calcium exchanger (NCX) [54,55], transient receptor potential (TRP) channels such as TRPA1 [56] and TRPC [57], among others. While *Itpr2*^−/−^ mice exhibit alternative mechanisms that are sufficient to support localized signaling, these are insufficient to drive global somatic responses.

In wild-type mice, astrocyte Ca^2+^ transients were not only integrated within each cell, but also highly synchronized across neighboring astrocytes, likely driven by neuromodulatory inputs, with noradrenaline (NA) playing a central role [58,59]. This widespread astrocytic activation suggests that neuromodulators may facilitate global synchronization of astrocytic activity during heightened behavioral states [27]. Such synchronized astrocytic responses could underlie coordinated regulation of cerebral blood flow, synaptic modulation, or broader network dynamics; however, the extent of these contributions remains to be fully elucidated [5,6,60]. In *Itpr2*^−/−^ mice, our data showed that this putative global coordination of astrocytic Ca^2+^ transients was notably reduced, with astrocytic Ca^2+^ activity appearing fragmented and restricted to small, isolated microdomains (Figure 2). Despite this profound reduction, when we calculated both WhiskMI and LocMI, we surprisingly found that these residue Ca^2+^ transients were still able to be modulated by both free whisking and locomotion. This suggests alternative pathways to initiate astrocytic Ca^2+^ signaling during locomotion, which could be closely linked to brain modulatory systems that may coordinate global network activity.

Unlike the data observed in astrocytes, the influence in the loss of IP3R2-dependent astrocytic Ca^2+^ on neurons was largely debated. One likely source of this discrepancy is the diversity of IP3R knockout models used across studies. Some utilized full IP3R2 knockouts (*Itpr2*^−/−^), while others target different isoforms or use conditional approaches, which can yield varying degrees of compensation by other signaling pathways [10,21,61]. Additionally, the brain region studied plays a major role; astrocytes in different areas may rely to different extents on IP3R2-dependent signaling. For instance, astrocytic modulation of hippocampal circuits might differ significantly from that in sensory cortex or the thalamus. Moreover, the type of behavioral tasks used can greatly influence outcomes. Tasks involving complex learning, memory, or stress responses may uncover deficits that simple locomotion or sensory tasks do not. Therefore, the apparent contradictions in the literature may reflect not only biological variability but also methodological differences in experimental design.

In our study, despite changes in astrocyte Ca^2+^ transients, neuronal activity in the vS1 was largely unaffected. Neuronal Ca^2+^ activity in *Itpr2*^−/−^ mice was similar to the ones in wild-type mice in both frequencies and amplitudes, echoing findings that IP3R2 deletion does not significantly affect baseline neuronal excitability [11,19,62]. Although all mice showed expected increases in neuronal activity during locomotion when compared to stillness, a slight reduction in event frequency and inferred spike rates during locomotion in *Itpr2*^−/−^ mice were observed (Figure 4B,C). In addition, the modulatory effect of locomotion on neurons was largely lost in *Itpr2*^−/−^ mice. These findings contribute to a broader and somewhat conflicting body of work on the functional consequences of IP3R2 deletion, particularly regarding neuronal activity and behavior. While some studies report little to no change in neuronal function or behavior in *Itpr2*^−/−^ mice [10,18,63], others have found more pronounced effects [20,22,24,64]. Our results add to this dialogue by showing that the brain modulatory effect on neurons might be affected by the absent of IP3R2, primarily manifesting as a marked reduction in astrocytic Ca^2+^transients. We speculate here that the reduction of astrocytic Ca^2+^ transients might be the major cause of the subtle impairment in neurons during locomotion, in which astrocytes might act as mediators to modulate neurons from large neuromodulatory inputs. This adds a new level of non-synaptic neuromodulation to the complexity of neuronal–astrocytic interaction. Further experiments directly involving elements of neuromodulation shall be considered to validate our above speculation.

In addition, astrocytes undergo pathological changes during ageing and age-related neurodegeneration, and they experience morphological changes and become reactive [65,66,67]. For instance, in Alzheimer’s disease (AD), these reactive astrocytes are observed around amyloid plaques, and their presence is related to cognitive decline [67,68,69,70]. Cortical astrocytic intracellular Ca^2+^ transients were also found elevated in anesthetized AD mice, implicating functional involvement of astrocytes in AD [71]. Our results offer a new mechanistic explanation of non-synaptic modulation in the participation of astrocytes via IP3R2 in brain cognition and the pathogenesis of various brain disorders. Our conclusion aligns to the previous-reported IP3R2 involvement in higher cognitive functions, such as remote behaviors and fear memory, which suggests that astrocytes might be involved in long-range synaptic connections and overall network function [72]. In animal models, studies also showed that *Itpr2*^−/−^ mice have been observed in various neurological and psychiatric disorders [73]. Thus, our suggested non-synaptic modulation via the astrocytic IP3R2 pathway may also provide future interests in developing novel therapeutic targets.

Another important point to consider in our study is how astrocytic Ca^2+^ signals were measured. In this study, we relied on manually drawn ROIs to quantify activity in different compartments, including soma, processes, glialpils, and endfeet. This approach allowed us to investigate spatial differences in signaling, but it has inherent limitations. Averaging fluorescence signals across relatively large regions can obscure fine-scale, localized events, particularly when activity is sparse or low in amplitude, as observed in *Itpr2*^−/−^ mice. Event-based analysis methods, such as AQuA (Astrocyte Quantitative Analysis) [74,75], offer an alternative strategy with fewer constraints than ROI-based approaches. Instead of relying on predefined regions, these methods detect and characterize Ca^2+^ events based on their intrinsic spatiotemporal features, enabling a more direct assessment of local activity patterns. This is particularly relevant given that Ca^2+^ signals typically emerge first in fine processes (microdomains) and later in the soma, a sequence that is difficult to resolve with static ROI. In our study, differences in onset timing across compartments were not readily apparent. One possible reason is that ROI-based analysis does not capture the true spatiotemporal kinetics of individual events. Additionally, since astrocytic compartments were identified based on the average GCaMP6f signal without an independent astrocytic marker, it is possible that some ROIs partially included signals from adjacent or out-of-focus structures, potentially blurring compartment-specific dynamics. Although we did not apply event-based analysis in the present study, future work using these approaches could help to better resolve the fine spatiotemporal organization of astrocytic Ca^2+^ signaling, particularly under conditions where signals are subtle or fragmented.

In summary, our study provides a non-synaptic modulatory perspective on functions of astrocytic Ca^2+^ transients to local neurons, particularly in IP3R2-dependent astrocytic Ca^2+^ elevation. The modulatory effect of locomotion on neurons were likely to be facilitated by local astrocytes and mediated by the coordinated high elevation of astrocytic Ca^2+^ during locomotion. In this case, the reduced astrocytic Ca^2+^ transients via IP3R2 were not affecting basic neuronal properties; however, they may potentially tune neuronal responses at the network level during animal behaviors.

## Data Availability

The original recorded data and original code for data analysis are available upon requests by the lead contact Wannan Tang (wannan.tang@ntnu.no).
